# Culturally adapted developmental outcome measure for Aboriginal and Torres Strait Islander children: study protocol for the validation of the ASQ-STEPS

**DOI:** 10.1136/bmjopen-2024-093029

**Published:** 2025-03-12

**Authors:** Anita D’Aprano, Cassie Boyle, Leah Lindrea-Morrison, Raelene Brunette, Emma Stubbs, Samantha Simpson, Patricia Eadie, Dan Cloney, Cuc Nguyen, Francesca Lami, Isabel Brookes

**Affiliations:** 1 Department of Paediatrics, Faculty of Medicine, Dentistry and Health Sciences, The University of Melbourne, Melbourne, Victoria, Australia; 2 Centre for Community Child Health, The Royal Children’s Hospital Melbourne, Melbourne, Victoria, Australia; 3 Larapinta Child and Family Centre, Northern Territory Government Department of Education, Alice Springs, Northern Territory, Australia; 4 Sunrise Health Service Aboriginal Corporation, Katherine, Northern Territory, Australia; 5 Public Health Division, Central Australian Aboriginal Congress, Mparntwe (Alice Springs), Northern Territory, Australia; 6 Faculty of Education, University of Melbourne, Melbourne, Victoria, Australia; 7 Australian Council for Educational Research, Camberwell, Victoria, Australia; 8 Melbourne Metrics, Faculty of Education, The University of Melbourne, Melbourne, Victoria, Australia

**Keywords:** Community child health, Psychometrics, Developmental neurology & neurodisability, Community-Based Participatory Research

## Abstract

**Abstract:**

**Introduction:**

Early childhood education and intervention programmes can improve the developmental outcomes for priority groups of children. However, in Australia, a culturally responsive developmental outcome measure that has been validated for use with Aboriginal and Torres Strait Islander children is required to effectively evaluate impact.

The Ages and Stages Questionnaire-Steps for Measuring Aboriginal Child Development (ASQ-STEPS) has been developed to fill this gap. A culturally responsive developmental outcome measure for children aged one to 60 months, the ASQ-STEPS is administered by interview and includes 52–63 items in each of five domains. This study aims to examine the reliability, validity, fidelity and acceptability of the ASQ-STEPS when used with Aboriginal and Torres Strait Islander children.

**Methods and analysis:**

This study uses a participatory approach, establishing an Indigenous Reference Group (IRG) and partnering with Aboriginal Community Controlled and government organisations. Up to 250 eligible children and their families will be recruited in urban, regional or remote locations in the Northern Territory, South Australia, Queensland, New South Wales and Victoria. Health or education practitioners will be engaged to administer the ASQ-STEPS. Demographic information, observational data during administrations, and caregivers’ and practitioners’ perspectives will also be collected.

Different analytical approaches will be employed to determine validity, fidelity and acceptability. A unidimensional item response model (IRM) will be fitted to the data by marginal maximum likelihood. Classical and IRM fit statistics and differential item functioning will be employed to examine validity. Internal consistency will provide an index of reliability and measurement error will assess the uncertainty associated with measures of individual children. Fidelity of administration will be observed and described, and acceptability and utility for caregivers and practitioners will be explored through thematic analysis.

**Ethics and dissemination:**

This study received ethical approval from the University of Melbourne and the ethical body in each site. Knowledge translation will be guided by the IRG. Results will be disseminated nationally and internationally.

**STRENGTHS AND LIMITATIONS OF THIS STUDY**

- Validating the first culturally responsive developmental outcome measure that was cocreated with Aboriginal and Torres Strait Islanders Community members.
- Using a participatory approach that promotes Aboriginal and Torres Strait Islanders Community members’ contribution to co-design and implementation of the research study. Of the 11 authors, 4 are Aboriginal (CB, LL-M, RB and ES).
- Including diverse Aboriginal and Torres Strait Islander voices in co-designing the developmental outcome measure.
- Barriers to timely recruitment of a sufficient sample of Aboriginal and Torres Strait Islander children and families.

## Introduction

 Research consistently supports the fostering of early childhood development for improved health and well-being across the lifespan.^1^ Studies have shown that early childhood learning and development programmes can improve the developmental and social outcomes of children experiencing adversity.^2 3^ To evaluate early childhood programmes and their impact on the learning and development outcomes of participating children, an effective developmental outcome measure is required.^4^ Developmental outcome measures can also help to determine the specific factors that contribute to improved outcomes, which may include characteristics of the programme and the participants.^5^,^8^ Experts recommend measuring child development outcomes as part of programme implementation.^8^

Aboriginal and Torres Strait Islander peoples have a continuing legacy of resilience, strength and determination, and most Aboriginal and Torres Strait Islander children grow up in nurturing environments connected to culture and community. However, not all Aboriginal and Torres Strait Islander children have such a strong start in life. The devastating effects of colonisation continue to have long-lasting impacts on health and well-being.^9 10^ Health disparities between Aboriginal and Torres Strait Islander and non-Indigenous children continue to exist, with the Australian Early Development Census reporting a decrease in Aboriginal and Torres Strait Islander children assessed as developmentally on track.^11^ Given the higher rates of developmental vulnerability faced by Aboriginal and Torres Strait Islander children, comprehensively measuring child development outcomes is imperative to ensure that Aboriginal and Torres Strait Islander children are receiving high-quality early childhood healthcare and education. To do this, however, services require an accessible, cost-effective, culturally responsive and valid developmental outcome measure.

Previous research has established the importance of culturally appropriate child development measures in the Aboriginal and Torres Strait Islander context.^12^,^15^ Applying standard child development measures with Indigenous populations without adaptation can be problematic and potentially harmful.^16^ Notably, this practice contradicts the needs expressed by Aboriginal and Torres Strait Islander communities, who have consistently called for culturally responsive and safe approaches to service provision. The National Australian and Torres Strait Islander Early Childhood Strategy^17^ clearly articulates the need for access to culturally safe primary and allied healthcare services, including ‘high-quality health and developmental assessments’ as one of the desired outcomes of the strategy. This aligns with the Aboriginal and Torres Strait Islander Health Plan 2021–2030^18^ and the overarching policy—the National Agreement on Closing the Gap^19^—which describe the fundamental need for embedding cultural safety values, behaviours, standards and resources within the workplace across health systems. There is little doubt that culturally responsive child developmental measures are required to optimise the quality and safety of developmental care Aboriginal and Torres Strait Islander children receive in Australia.

### Developmental outcome measures

Developmental outcome measures provide a comprehensive assessment of a child’s development across multiple domains. They can be used for programme evaluation, in order to evaluate the impact of childhood learning and development programmes on children’s development, and as population level measures of development. They can also be used across sectors, including education, health, research and policy. In contrast to developmental screening tools, such measures include enough items to more accurately reflect a child’s range of ability and can detect changes over time.^20^ While developmental screening tools, such as the Ages and Stages Questionnaires, 3rd Edition (ASQ-3),^21^ have been used as population monitoring and outcome measures,^22^ their utility for this purpose is limited: screening tools are not primarily designed to monitor a child’s developmental progress over time.^23^

Developmental screening tools have a limited number of items per age interval and provide a brief evaluation of a child’s development to help identify children with developmental difficulties.^23 24^ Developmental outcome measures, such as the Bayley Scales of Infant and Toddler Development^25^ and the Griffith Mental Development Scales,^26^ on the other hand, provide detailed information about the child’s full range of ability. These instruments have been used to measure child development outcomes in Australia. However, the use of these tools, particularly in rural and remote settings, is limited by the level of administrator training required,^24 27^ implementation costs and the lack of specific validation studies in the Aboriginal and Torres Strait Islander population. There is a significant gap in the availability of a developmental outcome measure that is accessible, cost-effective, psychometrically valid and culturally appropriate for Aboriginal and Torres Strait Islander children. The ASQ-Steps for Measuring Child Development (ASQ-STEPS) is a tool that is being adapted to fill this gap.

### The ASQ-Steps for Measuring Child Development (ASQ-STEPS)

**Figure 1:**
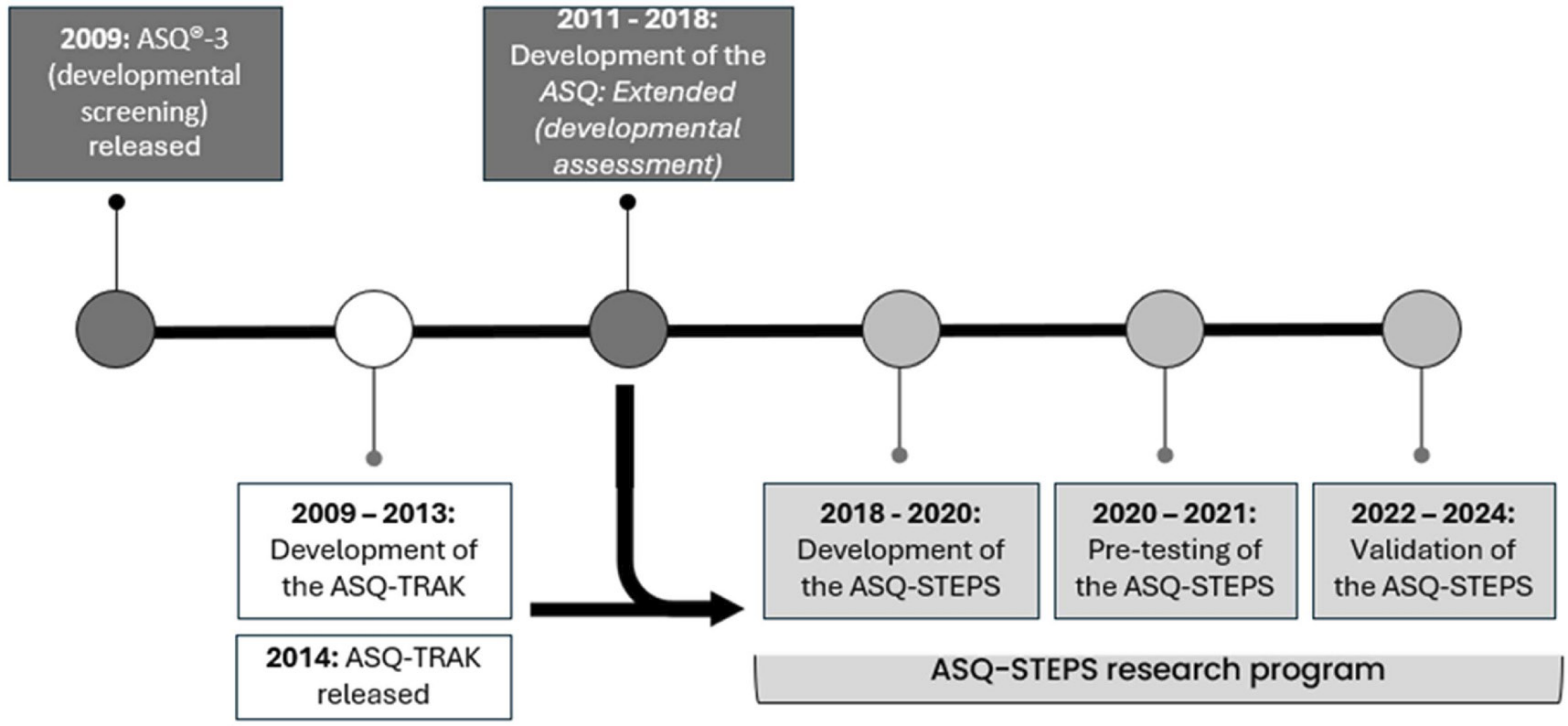
Phases of the Ages and Stages Questionnaire-Steps for Measuring Aboriginal Child Development (ASQ-STEPS) development.

The ASQ-STEPS has been developed as an outcome measure for Aboriginal and Torres Strait Islander children, synthesising the strengths of two pre-existing tools: the ASQ-TRAK^28^ (Ages and Stages Questionnaire-Talking about Raising Aboriginal Kids) and the *ASQ: Extended*^29^ (initially referred to as *ASQ: Inventory*). Figure 1 highlights the phases in the development of the ASQ-STEPS. The ASQ-TRAK is a developmental screening tool that has been adapted from the ASQ-3^21^ for its use with Aboriginal and Torres Strait Islander children aged 1 month to 5 ½ years.^30^ The *ASQ: Extended* is a single measure of child development for children aged 1 month to 3 years, created by the ASQ-3 authors and based on ASQ-3 items. The ASQ-STEPS combines the item content from the ASQ-3 and ASQ-TRAK, with the administration procedures of the *ASQ: Extended*.

ASQ-STEPS measures five domains, including communication, gross motor, fine motor, personal–social and problem-solving.^31^ However, while the ASQ-STEPS shares the same domains, many of the same items, and the same interview-style administration approach as the ASQ-TRAK, it differs significantly in its structure and number of items. The ASQ-TRAK makes available six items per domain for each of the 21 age intervals. In contrast, the ASQ-STEPS is not divided into age intervals and includes 52–63 items per domain, offering a more extensive range of items on a continuum.

The ASQ-STEPS was designed to be used with Aboriginal and Torres Strait Islander children and families in accordance with the International Test Commission on the cultural adaptation and translation of ability tests^32^ and the Toolkit for Measuring Childhood Development in Low- and Middle-Income Countries produced by The World Bank.^33^ A prototype of the ASQ-STEPS has been developed and undergone pretesting in an Australian urban and remote site.^31^

The primary aim of the current study is to explore the reliability and validity of the ASQ-STEPS as a measure of child development in the Aboriginal and Torres Strait Islander population.

The specific objectives of this research are as follows:

1. To investigate the reliability of the ASQ-STEPS domain measures and uncertainty associated with measures of individual children.
2. To investigate how well items measure the developmental domains by conducting item analysis of the ASQ-STEPS.
3. To investigate the construct validity of the ASQ-STEPS by evaluating whether the domain scores increase as a function of the child’s age and other child and family characteristics.
4. To investigate convergent validity by evaluating measurement non-invariance with the common items shared between ASQ-STEPS and other ASQ: Extended items.
5. To describe the fidelity of administration of the ASQ-STEPS, following practitioner participation in the training programme.
6. To investigate face validity by exploring utility and acceptability of the ASQ-STEPS among practitioners and caregivers.

## Methods and analysis

### Design and setting

The ASQ-STEPS validation study uses a participatory action research approach involving a collaboration between researchers, research participants and their community.^30^ A collaborative approach ensures that the research delivers benefit to the community and that the development, implementation and dissemination of the research is culturally appropriate.^34^ A collaborative approach is conceptualised as an iterative process that includes different phases and roles for members of the community.^35 36^ We acknowledge that while responsive to the need identified by communities, this study was originally conceptualised by non-Indigenous researchers, prior to the establishment of the formal Indigenous Reference Group (IRG). However, a number of factors ensure the centring of Aboriginal and Torres Strait Islander voice: the tool was co-created with Aboriginal and Torres Strait Islander people; the IRG will be involved in the design, implementation and translation phases; and the research team will be partnering with Aboriginal and Torres Strait Islander community members through the partnering community sites.

Sites will be located in urban, regional and remote areas in the Northern Territory, South Australia, Queensland, New South Wales and Victoria to capture the diversity of Aboriginal and Torres Strait Islander peoples. Site partners will include Aboriginal Community Controlled Health Services, Aboriginal Community Controlled Organisations, Government Health Services and Government Education Services. This study will be conducted between September 2022 and June 2025.

#### Governance

The ASQ-STEPS IRG consists of Aboriginal members who participated in the original development of the ASQ-STEPS,^31^ as well as other Aboriginal leaders in early childhood development, from health and education backgrounds. IRG members are supported by their organisation to participate but do receive an honourarium from the research team. Participating organisations and communities are invited to nominate a member for the ASQ-STEPS IRG to ensure all communities are represented. The IRG meets two times per year, face-to-face, for an all-day meeting in varying locations. The IRG centres the Aboriginal and Torres Strait Islander voice, and in accordance with a co-designed terms of reference ensures that the study is culturally appropriate and relevant. The IRG also provides overall guidance on approaches, such as to family recruitment, and assists with developing recruitment and informed consent materials. Finally, the IRG will codevelop the dissemination approach and cofacilitate the sharing of research findings with community members.

### Study sample

The study sample will include 250 children and their caregivers and up to 4 practitioners from each of the 6 sites. The sites may not be representative of the whole Aboriginal and Torres Strait Islander population. However, we aim to recruit a range of communities from diverse areas across Australia.

#### Practitioners

Due to typical qualification requirements for the administration of developmental assessments, practitioners with Allied Health, Early Intervention or Early Childhood Teaching qualifications will be invited to participate^24 27^. Practitioners will also have experience in early child development and administering the ASQ-TRAK in health or education settings.

#### Children and caregivers

The children will identify as Australian Aboriginal and/or Torres Strait Islander; be aged between 1 and 60 months; attend health or education programmes at the research sites; and have a parent, guardian or caregiver (hereafter caregiver) who consents to participate in the study. For the purpose of this study, the caregiver is defined as the adult who accompanies the child and identifies as their main carer and/or has a significant connection with the child. This is most likely to be the parent. However, Aboriginal and Torres Strait Islander cultural practices in family life reflect a complex and dynamic system, and the responsibility of child-rearing is not confined to the nuclear family.^37^ Therefore, this inclusive definition acknowledges the significant role that extended family members (aunts, uncles, cousins and grandparents) play in child-rearing in Aboriginal and Torres Strait Islander communities.^38 39^ The caregiver will need to speak some English as the ASQ-STEPS will be administered in Standard Australian English.

### Recruitment and consent

A volunteer sampling approach will be adopted to recruit children and their caregivers, and practitioners in the partnering sites to collect data related to objectives 1–5. Throughout the recruitment phase of this study, we will monitor enrolment to ensure that children and families with different characteristics will be enrolled.

A purposive sampling approach, an approach identified as appropriate for this context,^35 40^ will be adopted to further inform objective 6, the tool face validity. Furthermore, participating organisations will display posters to inform the community about the study. Posters will provide an overview and local contact information to encourage community members to find out more and/or express their interest in participating.

#### Practitioners

Practitioners will be provided with verbal information about the study as well as a plain language statement. Practitioners will be made aware that participation is voluntary and not participating in the study will not affect their work or position at the organisation.

#### Children and caregivers

As partners in the research process, participating organisations will identify eligible children attending their programmes. The Aboriginal Liaison Officer who is connected to the programme will contact caregivers. The Aboriginal Liaison Officer will inform the caregivers about the study and obtain their consent to be contacted via phone or approached face-to-face by the local Aboriginal Research Assistant employed for the study. If caregivers agree, the Aboriginal Research Assistant will provide verbal information face-to-face about the study at a location that suits the caregiver (i.e., at the child’s early education programme or an outreach visit). A pictorial booklet has been developed with the ASQ-STEPS IRG to support the Aboriginal Research Assistant in the recruitment of families to ensure caregivers understand what participation in the study involves. Caregivers will also be given a plain language statement explaining the purpose of the study, what is required of the participant and the risks and benefits of participation.

### Data collection

#### Measures

Table 1 describes the measures used in this study, in addition to the ASQ-STEPS. It describes their purpose in relation to the objectives and the intended sample size for their administration.

**Table 1:** Additional study measures to explore ASQ-STEPS administration fidelity and practitioners and caregivers’ perceptions of the ASQ-STEPS instrument

Measure		Administration		Participants and planned sample*	Purpose
	Mode	Items (n)	Time (min)		
Observation checklist	Face to face	35	90	12 practitioners†	To describe the practitioners’ ASQ-STEPS administration, scoring and feedback procedures. This will address the fidelity by assessing the degree to which the practitioner performs tasks according to the competencies established during the training (objective 5).
Caregiver questionnaire	Face to face	14	15	250 caregivers	To collect caregiver’s demographic information‡, perceptions of the ASQ-STEPS instrument, and their experience completing the assessment with their child. This will provide data from all caregivers on the acceptability and utility of the ASQ-STEPS (objective 6).
Caregiver semistructured interview schedule	Face to face*	5	15–30	25 caregivers	To explore the perspectives of caregivers on the acceptability and utility of the ASQ-STEPS in more depth. This will inform the final version of the ASQ-STEPS and will also inform the approach, including training of practitioners in the implementation of the tool (objective 6).
Practitioner questionnaire	Online survey	11	10	12 practitioners†	To collect practitioners’ perceptions of the content, administration approach, and value of the ASQ-STEPS (objective 6).
Practitioner semistructured interview schedule	Face to face*	7	15–30	12 practitioners†	To explore the perspectives of practitioners on the acceptability and utility of the ASQ-STEPS in more depth. This will inform the final version of the ASQ-STEPS and will also inform the approach, including training of practitioners in the implementation of the tool (objective 6).

* To facilitate data collection, administration may occur via teleconference or phone call.

† The number of practitioners is assumed to be up to four per site, with an anticipated average of two practitioners per site. Observations of practitioners will be performed for 10% of the administrations of the tool in each site, including at least one observation per practitioner. All practitioners will be invited to respond to questionnaires and will be invited to participate in the interviews.

‡ It includes data on caregiver level of education, health care card status, address, number of children in the home and employment status

ASQ-STEPSAges and Stages Questionnaire-Steps for Measuring Aboriginal Child Development

##### Ages and Stages Questionnaire-Steps for Measuring Aboriginal Child Development (ASQ-STEPS)

The ASQ-STEPS developmental outcome measure assesses children’s skills in five domains of development (see below). Structured as a single measure, each domain includes a range of items spanning developmental abilities from 1 to 60 months of age. All items are written in plain English and illustrated to promote caregiver understanding and engagement.

The practitioner administers the ASQ-STEPS in an interview style, with the caregiver promoting a family-centred, strength-based approach. The items ascertain a child’s ability on a developmental continuum: gross motor (60 items); fine motor (61 items); communication (52 items); problem-solving (63 items); and personal social (58 items). The range of items in each developmental domain allows for multiple administrations to monitor children’s developmental progress and evaluate programme impacts on a child’s development. Item response options are ‘yes’ (2 points) for mastered skills, ‘sometimes’ (1 point) for emerging skills and ‘not yet’ (0 points) for skills yet to develop.

A basal and ceiling approach is applied to limit the number of items administered with the child. The starting point for each domain is determined based on the child’s age. To obtain the basal score, a child must score ‘yes’ for five consecutive items. To obtain the ceiling score, a child must score ‘not yet’ for five consecutive items.

The ASQ-STEPS instrument also collects demographic information including child name, date of birth, gender, gestation, languages spoken with child, caregiver name, and caregiver relationship to child.

### Procedures

**Figure 2:**
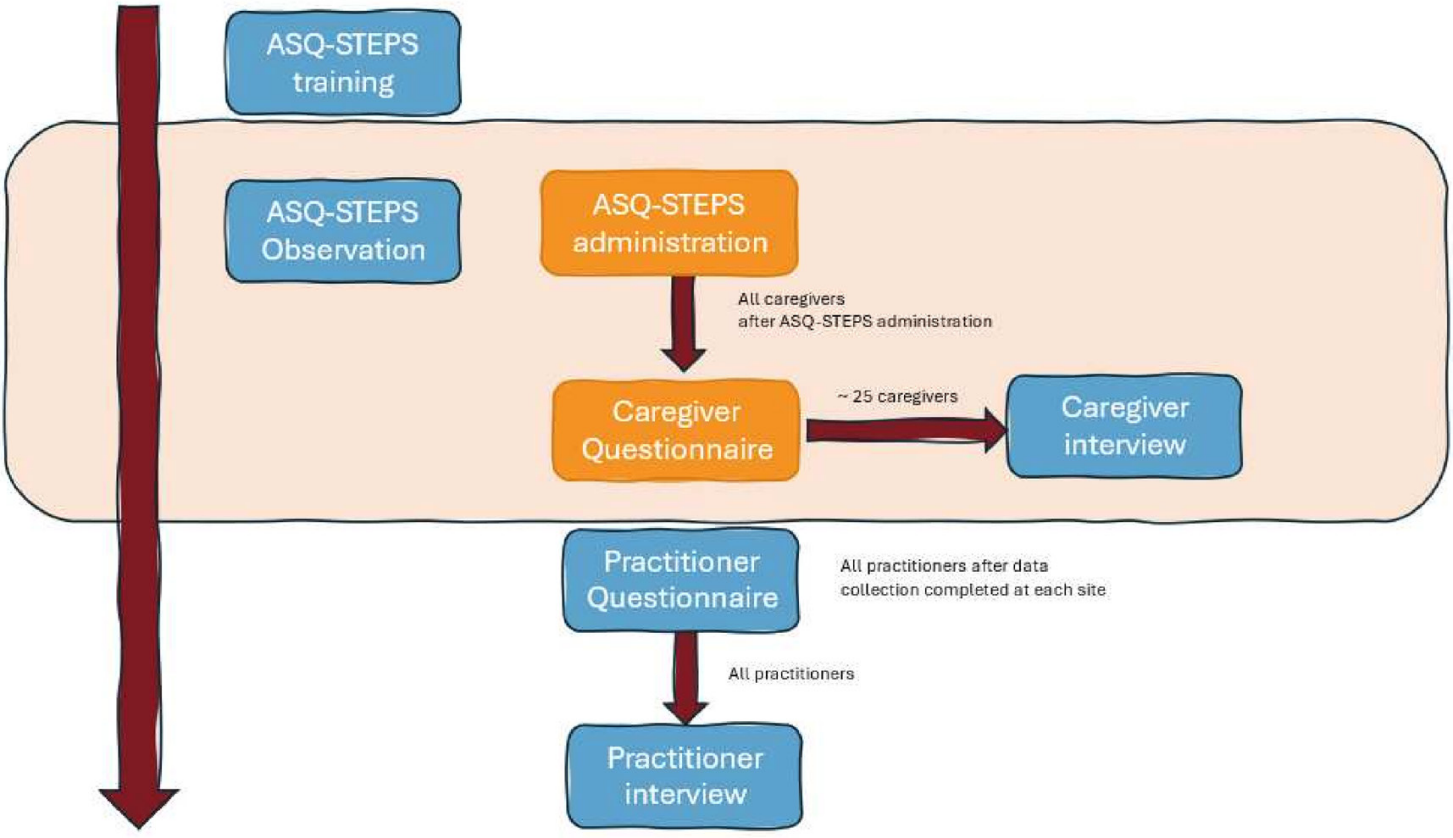
Procedures for data collection. Tasks in orange boxes are performed by staff at each site with children and their caregivers. Tasks in blue boxes are performed by the research team.

Figure 2 illustrates the procedures for data collection, described below.

#### ASQ-STEPS training

Practitioners and support staff will attend formal ASQ-STEPS training, developed and delivered by the research team, and derived from and aligned with the evidence-based ASQ-TRAK training.^41^ Training will be delivered over 1 day on-site. The training includes content on the development of the ASQ-STEPS instrument, the family-centred, culturally responsive administration approach, scoring and providing feedback to caregivers in a strength-based manner. It is a requirement that practitioners have already completed ASQ-TRAK training due to the similarity in the style of administering the tool and the importance of considering the cultural safety of families. This serves as a professional development opportunity for staff, who may use either the ASQ-STEPS or the ASQ-TRAK as a component of their work in the future.

#### ASQ-STEPS administration

Caregivers will be asked to attend their local health or education service with their child at a prearranged time for an ASQ-STEPS appointment. A local Aboriginal Family Support Worker recruited for the study will support attendance and administration.

Trained practitioners will administer the ASQ-STEPS instrument with 10–20 children in partnership with their caregivers, over a period of approximately 3 months. The final number will depend on the number of participating practitioners and local child eligibility. The practitioner will begin the administration by introducing the ASQ-STEPS instrument, completing the child and caregiver demographic information, and explaining the process, including the administration process and scoring options, and introducing each domain. The items are presented to the caregiver using a visual flip chart to optimise engagement. The flip chart includes a colour illustration of the item and short explanations of the skill being targeted, as well as scoring and administration guidance for the practitioner.

As an interview-style assessment, caregivers are asked if they have observed their child perform a certain skill that pertains to one of the five developmental domains. The caregiver is then encouraged to support the child to demonstrate every skill, while the practitioner co-observes. On each item, the caregiver and practitioner determine if a response of ‘yes’, ‘sometimes’ or ‘not yet’ is scored. Administration of the ASQ-STEPS is expected to take up to 90 min. If required, the ASQ-STEPS can be administered over two sessions in a single week. A paper scoring booklet will be used to record the response to each item administered and any additional notes. To ensure developmental feedback is provided to the families in a meaningful way, the practitioner will provide caregivers with the results of their child on the ASQ-STEPS by mapping scores for each domain to the ASQ-TRAK developmental screening tool,^30^ with appropriate recommendations. If the child scores below the cut-off in any of the five developmental domains, they will be referred to and supported to attend the appropriate service for further assessment.

Each child and caregiver will be assigned a unique identifier during data entry. The de-identified data will be entered into a REDCap^42^ database developed by the research team.

#### Observation

The research team will observe ASQ-STEPS administrations with children across several different age ranges and with each of the practitioners (up to 10% total administrations). Using a purpose-designed observation checklist (measure A, table 1), researchers will observe practitioners’ administration. The observation checklist, derived from the competency checklist used in the ASQ-TRAK training,^41^ records information on all areas of the application of the ASQ-STEPS administration approach (preparation, introduction, item administration, scoring and feedback). For this study, the observation will be used to explore fidelity, rather than used to provide feedback to the practitioner. Field notes will also be taken on the paper form. The research team will enter these data into the REDCap database.

#### Caregiver questionnaire

Following the ASQ-STEPS appointment, all caregivers will be invited to complete a questionnaire (measure B, table 1) with the support of the practitioner or the local Aboriginal Research Assistant. Responses will be recorded on paper and then scanned and entered into the REDCap database.

#### Caregiver semistructured interview schedule

A proportion of participating caregivers (10%) will be invited to participate in a semistructured interview (measure C, table 1). The interviews will be conducted face to face by an Aboriginal Research Assistant at the service or caregiver nominated environment. Interviews will be completed within 1 week of the caregiver’s participation in the ASQ-STEPS. The interview will be audio recorded and transcribed (with caregiver consent). If the caregiver would prefer the interview not to be recorded, then hand-written notes will be taken.

#### Practitioner questionnaire

Following the data collection period, all practitioners will complete a short online questionnaire (measure D, table 1). The questionnaire will take approximately 10 min to complete, and practitioners’ responses are anonymous. Questionnaire data will be recorded electronically using Qualtrics.^43^

#### Practitioner semistructured interview schedule

All practitioners will participate in an individual semistructured interview (measure E, table 1) conducted by a member of the research team. Interviews will be completed on the telephone or via a video conferencing platform (e.g., Zoom). The interview will be audio-recorded and transcribed (with practitioner consent). If the practitioner would prefer the interview not to be recorded, then hand-written notes will be taken.

### Analytic approach

Different analytical approaches will be adopted to address each of the objectives.

To investigate the reliability of the ASQ-STEPS, we will assess the person separation reliability index. This is the most important index demonstrating the extent to which the tools truly separate children by their capabilities. Measurement error will be calculated to assess the uncertainty associated with measures of the sample and individual children. The internal consistency by measuring Cronbach’s alpha will also be reported.

This study will investigate if the data collected using ASQ-STEPS fit item response models (IRMs) to assess whether linear measures (stages of developmental progression) can be developed to strengthen reporting. To investigate the extent to which items contribute to the construct of each development domain of the ASQ-STEPS, a polytomous scored unidimensional IRM^44^ will be fitted to the data using the TAM package^45^ of open-source statistical software programme R.^46^ A typical item analysis will be conducted, including assessment of classical and IRM test statistics including Item Facility, item-rest correlation, point-biserial correlation, fit statistics (including item characteristic curves), ordering of category average abilities, item thresholds’ implied order (compared with published or intended order) and dimensionality (by standardised residuals and design-implied fit).^47^ Test targeting will be reviewed by test information functions, Wright map, comparison between the mean of item difficulties and mean of children’s abilities and whether there are floor or ceiling effects.

To test the construct validity of the ASQ-STEPS, we will calculate measurement non-invariance through differential item functioning. Differential item functioning will be assessed by child’s age, sex, maternal level of education, number of children in the home, socio-economic status, employment status and remoteness.

Differential item functioning analysis will also be conducted between the Australian data and international data related to the *ASQ-3: Extended* to evaluate the extent to which the tools behave the same way across two populations. The latent variance–covariance matrix will also be calculated to understand the extent to which dimensions overlap.

To describe fidelity of administration, we will calculate the proportion of practitioners who follow or partially follow each step (or competency) from the observation checklist. We will adopt Braun and Clarke’s reflexive thematic analysis approach^48^ to analyse the qualitative data from descriptive field notes and transcripts from the practitioner and caregiver interviews. The research team will transcribe field notes and audio recordings, which will be uploaded into NVivo qualitative software^49^ to facilitate coding. The IRG members and Aboriginal Research Assistants, together with the research team, will develop a coding topic guide for the initial analysis. Line-by-line coding of data will be conducted, and initial nodes will be refined and expanded through iterative coding. Candidate themes will be developed and finalised through a consensus meeting between the authors and the IRG members and Aboriginal Research Assistants.

#### Sample size considerations

We aim to recruit 250 children. While a sample greater than 250 individuals is optimal to perform IRT model fit statistics,^32 50^ recruitment of Aboriginal and Torres Strait Islander children and families in studies on child development presents challenges. This is a small population, often living in remote areas, and there are further barriers related to historical and current contexts, including distrust of the research process resulting in difficulty engaging families.^2851^,^53^ This sample size does, however, meet the criteria for the adequate number of participants required^50^ to perform linear model fit statistics analysis to validate that the instrument accurately reflects the underpinning developmental progression, while ensuring timely completion of this study. Previous studies assessing the face validity of measures adapted for Aboriginal and Torres Strait Islander children and families^54^,^56^ informed the sample size required to reach data saturation for the qualitative analysis.

## ETHICS AND DISSEMINATION

### Ethics

The study received ethical approval from the University of Melbourne (Application ID 23688) and from the appropriate ethical body in each site (Northern Territory Human Research Ethics Committee: 2020-3929; Aboriginal Health & Medical Research Council of NSW: 2150/23; Page 20 of 26 Queensland Health: HREC/2019/QTHS/56008). Approval was also received from organisational research units where additional ethical approval was not required. We do not have ethical approval to name participating sites.

The study demonstrates a commitment to the NHMRC guidelines ^57^ of cultural continuity, equity, reciprocity, respect, and responsibility, all of which are bound together by the core central value of spirit and integrity. By working in a collaborative partnership with communities who have recognised early childhood development as a priority and chosen to participate in the research, there are several benefits for Aboriginal and Torres Strait Islander people and communities. Reciprocal benefits include the resulting tool that can be used to measure the impact of early childhood learning and/or intervention programs for Aboriginal and Torres Strait Islander children and direct funding to programs that have demonstrated effectiveness, and capacity-building for local practitioners who provide child health services.

### Dissemination

A comprehensive dissemination strategy has been developed for the ASQ-STEPS validation study, by the Aboriginal Research Assistant employed on the study, the IRG and the research team. Strategies will include accessible and culturally appropriate methods, such as face-to-face community visits, email, paper-based materials, and other research outputs such as websites, conference presentations and peer reviewed publications, to reach a diverse range of stakeholders at the local community, national, and international level. IRG members will lead the knowledge translation activities in Community, which will include providing feedback to caregivers for consideration, and reporting to the service providers who were part of the process. Community partners and IRG members will be included as co-authors on conference papers and publications
