# Supplementary material for: Culturally adapted developmental outcome measure for Aboriginal and Torres Strait Islander children: study protocol for the validation of the ASQ-STEPS
Source: BMJ Open. 2025 Mar 12;15(3):e093029. doi: 10.1136/bmjopen-2024-093029 (PMC11904345; doi:10.1136/bmjopen-2024-093029)
Supplement: Supplementary file 1 [file bmjopen-2024-093029.reviewer_comments.pdf]

PEER REVIEW HISTORY

BMJ Open publishes all reviews undertaken for accepted manuscripts. Reviewers are asked to complete a checklist review form and are provided with free text boxes to elaborate on their assessment. These free text comments are reproduced below.

ARTICLE DETAILS

Title (Provisional)

Culturally adapted developmental outcome measure for Aboriginal and Torres Strait Islander children: study protocol for the validation of the ASQ-STEPS.

Authors

D'Aprano, Anita; Boyle*, Cassie; Lindrea-Morrison*, Leah; Brunette*, Raelene; Stubbs*, Emma; Simpson, Samantha; Eadie, Patricia; Cloney, Dan; Nguyen, Cuc; Lami, Francesca; Brookes, Isabel

---

VERSION 1 - REVIEW

**Reviewer**	**1**
**Name**	**Shepherd, Carrington**
**Affiliation**	**Telethon Kids Institute**
**Date**	**11-Oct-2024**
**COI**	**None**

This paper is a research study protocol for a newly developed measure of Aboriginal and Torres Strait Islander child development. The paper is well written, with clearly defined aims and objectives and, to my way of thinking, satisfies the conditions of a robust protocol for a much-needed project.

I have only a few, minor comments for consideration:

- My main issue is that I believe the paper could provide a fuller articulation of the reasons why existing measures of child development aren't necessarily suitable for Aboriginal children—that is, why they aren't (i) necessarily measuring what communities see as important markers of child development or (ii) accurately measuring the construct of interest. This was well-considered and documented in an Australian Early Development Index (AEDI) Indigenous Adaptation Study (2009; see reference, below). It would seem important to discuss this in the Introduction section, especially around lines 16-23 (page 4) where the AEDC is mentioned.

- Abstract: if possible within the word limit, I would add a few more details – namely, which state/territories the project will be run in; what type of sites and practitioners are involved; and some more description of the tool (number of questions, how administered, who the respondent(s) is(are)).

- Abstract: the intro mentions the tool is for 1-3 year-olds but elsewhere references 1-60 months?

- Page 4 (lines 26-27): Could also mention that a measure can be an important population level measure of development?

- Page 5 (line 18): I think valid is a better word than tested here (we want measures that work)

- Page 6 (line 15): has the tool/measure already been adapted for use with Aboriginal children specifically? Later in that para: which urban and remote sites (Australia or elsewhere?)?

- Page 9 (line 11): who are the participating organisations (types)?

- Page 9 (line 13): any adaption in Aboriginal English?

- Page 13 (line 18): what is the process if there is disagreement between the practitioner and the caregiver (would seem a likely common event)?

- Page 14 (line 11): is the observation process used to continually improve practitioner performance?

- Page 14 (line 17) to Page 15 (line 17): would be worthwhile, for each of these sections, to describe what these questionnaires/interviews are collecting/asking.

- Page 17: I think the justification for the sample size is probably robust enough, although no calculation is done. Should there be?

- Page 17 (line 10): an international reader may not understand what these barriers are.

- Page 18 (line 19): assume these translation activities involve taking results back to the community for consideration, including to service providers who were part of the process?

- Page 18 (line 25): is there a reference for pre-testing study? Also, there is an asterisk in this para but not sure what it refers to.

Reference:

Silburn, S., Brinkman, S., Ferguson-Hill, S., Styles, I., Walker, R. and Shepherd, C., 2009. The Australian early development index (AEDI) indigenous adaptation study. Perth: Curtin University of Technology and Telethon Institute for Child Health Research.

**Reviewer**	**2**
**Name**	**Gladstone, Melissa**
**Affiliation**	**University of Liverpool, Department of Women and Children's Health, Institute of Life Course and Medical Sciences**
**Date**	**23-Oct-2024**
**COI**	**None**

This protocol paper describes a study which aims to look at the ASQ-STEPS – a new tool based on the ASQ TRAK and the ASQ Extended – for use in Aboriginal Australian settings. The team have used the International Test Commission advice on cultural adaptation and translation of tests as well as some advice from the World Bank Toolkit. It may also be useful to just check through the guidelines provided by COSMIN which provide excellent information on how to create quality validity studies.

The primary aim of this study is to look at the validity and reliability of the tool. The objectives include conducting item level analysis, construct validity (as a function of age) and convergent validity (comparing non-variance of similar items in ASQ Extended), fidelity (by practitioners) and face validity (utility and acceptability). The team demonstrate great thought in ensuring that the process is co-driven by Aboriginal and Torres Islander representatives.

It is surprising that the study team do not discuss the fact that there are many tools that have been created for use in LMIC settings that have good validity and reliability. This includes a number of tools which have been validated across a number of countries – including the WHO GSED,

the CREDI, the WHO IYCD, the GMCD and the RNDA – as well as tools such as MDAT and the Kilifi Developmental Inventory. It would be important to understand why they did not use or consider any of these tools or the evidence gathered from the creation of these tools in the work they did on Ages and Stages.

A purposive approach is being taken to identify 250 children from 0-6 to take part in the study. 250 children seems a limited number to be able to assess the construct validity across age. I can see this has been discussed but it is quite a big limitation of the study.

It is not clear as to whether the researchers are concerned at all about ensuring that there is a random selection of children from a representative sample of the population – rural vs urban, low SES vs high SES, children more at risk or less at risk. The validity of the tool may be less so if the selection of participants is not random. Do the authors discuss this and/or discuss the sample size which seems low for this type of study (unless conducted longitudinally). Presently they are only collecting information on date of birth, gender, gestation, languages spoken with child, caregiver name, and caregiver relationship to the child. Will they collect information on socioeconomic status, rural vs urban, risk factors in the family, nutritional status of child, any health factors? This would be important in considering construct validity.

The team are conducting reliability by looking at within tool reliability. It would be helpful to understand why the team did not choose to do inter-rater or intra-rater reliability of the tool to understand how well it is functioning when used by the same or other practitioners who might be using the tool. I may have missed that the team were doing this?

The team might want to consider defining more carefully who the caregiver is – and will they ensure that they are consistent as to who they consider the caregiver in the study – this is likely to affect results.

I was surprised that the tool takes 90 minutes. This seems a long time to conduct a developmental test – particularly when it is a parent reported tool. It would be good to discuss this.

This study should be reviewed by a statistician who works in psychometrics who can review the plans for IRT analysis.

Qualitative pieces of work – I wondered if the team were considering using any specific frameworks for understanding the themes coming from their results? Consideration of a framework for acceptability (Sekhon can be helpful) or feasibility might be helpful to provide some structure for a topic guide and possibly for some deductive thematic analysis which might also be inductive alongside. I was unclear what they then plan to do with this data in terms of the ASQ-STEPS – are they mainly looking at the way that the tool is implemented or are they looking at items within the tool?? Or other areas? E.g. feedback. Time taken, ethics etc etc….

VERSION 1 - AUTHOR RESPONSE

Journal BMJ Open<br>Title **Culturally adapted developmental outcome measure for Aboriginal and Torres Strait Islander children: study protocol for the validation of the ASQ-STEPS.**<br>ID- bmjopen-2024-093029	
Reviewer's comment to authors	Authors' response
Reviewer 1	
This paper is a research study protocol for a newly developed measure of Aboriginal and Torres Strait Islander child development. The paper is well written, with clearly defined aims and objectives and, to my way of thinking, satisfies the conditions of a robust protocol for a much-needed project.<br><br>I have only a few, minor comments for consideration:	Thank you for your considered comments.
1.1 My main issue is that I believe the paper could provide a fuller articulation of the reasons why existing measures of child development aren't necessarily suitable for Aboriginal children—that is, why they aren't (i) necessarily measuring what communities see as important markers of child development or (ii) accurately measuring the construct of interest. This was well-considered and documented in an Australian Early Development Index (AEDI) Indigenous Adaptation Study (2009; see reference, below). It would seem important to discuss this in the	Thank you for pointing this out. We have added a paragraph following the section where the AEDC is mentioned:<br><br>"Previous research has established the importance of culturally appropriate child development measures in the Aboriginal and Torres Strait Islander context [12-16]. Applying standard child development measures with Indigenous populations without adaptation can be problematic and potentially harmful [17]. Notably, this practice contradicts the needs expressed

Introduction section, especially around lines 16-23 (page 4) where the AEDC is mentioned.	by Aboriginal and Torres Strait Islander communities, who have consistently called for culturally responsive and safe approaches to service provision. The National Australian and Torres Strait Islander Early Childhood Strategy [18] clearly articulates the need for access to culturally-safe primary and allied health care services, including "high-quality health and developmental assessments" as one of the desired outcomes of the strategy. This aligns with the Aboriginal and Torres Strait Islander Health Plan 2021-2030 [19] and the overarching policy - the National Agreement on Closing the Gap [20]- which describe the fundamental need for embedding cultural safety values, behaviours, standards and resources within the workplace across health systems. There is little doubt that culturally responsive child developmental measures are required to optimise the quality and safety of developmental care Aboriginal and Torres Strait Islander children receive in Australia."
1.2 Abstract: if possible within the word limit, I would add a few more details – namely, which state/territories the project will be run in; what type of sites and practitioners are involved; and some more description of the tool (number of questions, how administered, who the respondent(s) is(are)).	Thank you for highlighting additional information that would be of value in the Abstract for this paper. We are restricted by the word limit but have made the below changes to address this, including clarifying the inclusion of urban sites:<br><br>*"**Introduction.** Early childhood education and intervention programs can improve the developmental outcomes for priority groups of children. However, in Australia, a culturally ~~appropriate~~ responsive developmental outcome measure that has been validated for use with Aboriginal and Torres Strait~~s~~ Islander children is required to effectively evaluate ~~the~~ impact ~~of these programs~~.*<br><br>*The ASQ-Steps for Measuring Aboriginal Child Development (ASQ-STEPS) has been developed to fill this gap. A culturally responsive developmental outcome measure for children aged ~~1~~ one to ~~3 years~~ 60 months ~~of age~~, the ASQ-STEPS is administered by interview and includes 52 – 63 items in each of five domains. This study aims to examine the reliability, validity, fidelity and acceptability of the ASQ-STEPS when used with Aboriginal and Torres Strait Islander children.*

	Methods and analysis. *This study uses a participatory approach ~~through the establishment of~~ establishing an ~~advisory~~ Indigenous Reference Group ~~(IRG)~~ and partnering with ~~6 sites~~ Aboriginal Community Controlled and government organisations ~~which offering services to Aboriginal and Torres Strait Islander children and families living in urban, regional rural or remote locations~~. Up to ~~The sites will recruit a total of approximately~~ 250 eligible children ~~(age range 1-60 months)~~ and their families will be recruited in urban, regional or remote locations in the Northern Territory, South Australia and New South Wales. ~~On average 2~~ Health or education practitioners will be ~~employed~~ engaged ~~at each site~~ to administer the ASQ-STEPS. Demographic information, observational data during administrations, and caregivers' and practitioners' perspectives will also be collected."*
1.3 Abstract: the intro mentions the tool is for 1-3 year-olds but elsewhere references 1-60 months?	Thank you for identifying this potentially confusing inconsistency. The ASQ-STEPS is expected to ultimately be validated for use with children aged 1 to 42 months old. However, for the purpose of the validation study we have extended the age range of children to be included up to 60 months given the ASQ-STEPS instrument contains culturally modified ASQ-3 items developed for children up to 66 months of age. This will provide important information about the items for older children, including at what point we can anticipate children aged 36 - 42 months will reach their ceiling with current administration rules.<br><br>To reduce potential confusion, we have made the age range for the ASQ-STEPS consistent throughout the paper (1 - 60 months).
1.4 Page 4 (lines 26-27): Could also mention that a measure can be an important population level measure of development?	Thank you for this suggestion. We have added this to now read:<br><br>"They can be used for program evaluation, in order to evaluate the impact of childhood learning and development programs on children's development, and as population level measures of development. They can also be used across sectors…"
1.5 Page 5 (line 18): I think valid is a better word than tested here (we want measures that work)	Thank you for this suggestion. We have modified the wording to include "valid".

1.6 Page 6 (line 15): has the tool/measure already been adapted for use with Aboriginal children specifically? Later in that para: which urban and remote sites (Australia or elsewhere?)?	We have now provided more information to clarify these points.<br><br>"The ASQ-STEPS was designed to be used with Aboriginal and Torres Straits Islander children and families … A prototype of the ASQ-STEPS has been developed and undergone pre-testing in an Australian urban and remote site."
1.9 Page 9 (line 11): who are the participating organisations (types)?	In the preceding section (page 8, line 3 of original submission) we indicate the types of participating organisations.<br><br>"Site partners will include Aboriginal Community Controlled Health Services, Aboriginal Community Controlled Organisations, Government Health services, and Government Education services."
1.10 Page 9 (line 13): any adaption in Aboriginal English?	The items on the ASQ-STEPS (and ASQ-TRAK) have been modified to be in plain English, but have not been adapted into Aboriginal English. We have modified the section to read:<br><br>"The caregiver will need to speak some English as the ASQ-STEPS will be administered in Standard Australian English."
1.13 Page 13 (line 18): what is the process if there is disagreement between the practitioner and the caregiver (would seem a likely common event)?	We have made some changes to this section to highlight that every item is demonstrated, so that both the caregiver and the practitioner can observe the skill being performed. This provides the opportunity for a clear response.<br><br>"The caregiver is then encouraged to support the child to demonstrate ~~the~~ every skill ~~with the support of their caregiver~~, while the practitioner co-observes."
1.14 Page 14 (line 11): is the observation process used to continually improve practitioner performance?	The purpose of the Observation is to inform our understanding of fidelity of administration, and explore some of the barriers to fidelity.

	We will not have the opportunity to observe all the ASQ-STEPS so there will be little opportunity for this to be a Quality Improvement step.<br><br>We have amended this section to read:<br><br>"…researchers will observe practitioners' administration ~~fidelity~~. The Observation Checklist, derived from the competency checklist used in the ASQ-TRAK training [42] , records information on all areas of the application of the ASQ-STEPS administration approach (preparation, introduction, item administration, scoring and feedback). For this study, the Observation will be used to explore fidelity, rather than used to provide feedback to the practitioner. ~~This tool is derived from the competency checklist used in the ASQ-TRAK training~~"
1.15 Page 14 (line 17) to Page 15 (line 17): would be worthwhile, for each of these sections, to describe what these questionnaires/interviews are collecting/asking.	Thank you for this suggestion. We have included this information in table 1 in the purpose column.
1.16 Page 17: I think the justification for the sample size is probably robust enough, although no calculation is done. Should there be?	Thank you for your thoughtful point. We followed the recommendation from the COSMIN group for adequate number of participants required when applying IRT fit model statistics to study the validity of a test, as indicated by the reference in this section. Considering that there is no formal calculation, we opted to modify the title of this section to be "Sample size considerations" instead of "Sample size calculation".
1.17 Page 17 (line 10): an international reader may not understand what these barriers are.	We have edited this section to read:<br><br>"We aim to recruit 250 children. While a sample greater than 250 individuals is optimal to perform IRT model fit statistics [33, 50], recruitment of Aboriginal and Torres Strait Islander children and families in studies on child development presents challenges. This is a small population, often living in remote areas, and there are further barriers related to historical and current contexts, including distrust of the research process resulting in difficulty engaging families [29, 51-53]."
1.18 Page 18 (line 19): assume these translation activities involve taking results back to the community for consideration, including to service providers who were part of the process?	We have further clarified this section to read:

	"Strategies will include accessible and culturally appropriate methods, such as face-to-face community visits ~~contact~~, email, paper-based materials, and other research outputs such as websites, conference presentations and peer reviewed publications, to reach a diverse range of stakeholders at the local community, national and international level. IRG members will ~~join~~ lead the knowledge translation activities in Community, which will include providing feedback to caregivers for consideration, and reporting to the service providers who were part of the process."
1.19 Page 18 (line 25): is there a reference for pre-testing study? Also, there is an asterisk in this para but not sure what it refers to.	Thank you for the careful review of the manuscript. We have now referenced the pre-testing paper, as it is has recently been published.<br><br>The asterisk in this section was a footnote to the asterisk in the author list, identifying Aboriginal authors. We have removed the asterisk in the Acknowledgement section and added the explanation for the asterisk in the author affiliation section:<br><br>"*Aboriginal co-authors."
Reviewer 2	
This protocol paper describes a study which aims to look at the ASQ-STEPS - a new tool based on the ASQ TRAK and the ASQ Extended - for use in Aboriginal Australian settings. The team have used the International Test Commission advice on cultural adaptation and translation of tests as well as some advice from the World Bank Toolkit. It may also be useful to just check through the guidelines provided by COSMIN which provide excellent information on how to create quality validity studies.<br>The primary aim of this study is to look at the validity and reliability of the tool. The objectives include conducting item level analysis, construct validity (as a function of age) and convergent validity (comparing non-variance of similar items in ASQ Extended), fidelity (by practitioners) and face validity (utility and acceptability). The team	Thank you for your considered comments.

demonstrate great thought in ensuring that the process is co-driven by Aboriginal and Torres Islander representatives.	
2.1. It is surprising that the study team do not discuss the fact that there are many tools that have been created for use in LMIC settings that have good validity and reliability. This includes a number of tools which have been validated across a number of countries – including the WHO GSED, the CREDI, the WHO IYCD, the GMCD and the RNDA – as well as tools such as MDAT and the Kilifi Developmental Inventory. It would be important to understand why they did not use or consider any of these tools or the evidence gathered from the creation of these tools in the work they did on Ages and Stages.	Thank you for this comment. There are several reasons for why we did not choose to use a tool that has been created for LMIC settings. First, the Aboriginal and Torres Strait Islander context is quite a unique situation because although many of the same challenges that exist in LMIC settings are faced in community, this population is in Australia – a high income country. So we would not automatically explore tools that are used in LMIC.<br><br>Second, the Ages and Stages Questionnaires are used by many mainstream services across Australia. This was the main reason it was chosen as the tool to adapt to create the original ASQ-TRAK developmental screening tool. Having an adapted version of a tool that is already used in Australia facilitates implementation. The detail for this process is outlined in reference number 31 (formerly *25*), reported also below for your convenience.<br>Third, once we had the ASQ-TRAK tool creating an outcome measure by extending the developmental screening tool that had evidence for acceptability and validity seemed a logical choice. Finally, the ASQ authors were very supportive and open to collaborating on an adaptation for the Aboriginal context.<br><br>Furthermore, we were seeking a tool that could be used for individual child assessment. The GSED, CREDI and IYCD are all population measures and not validated for assessment of individual children.<br><br>D'Aprano A, Silburn S, Johnston V, et al. Adaptation of the Ages and Stages Questionnaire for Remote Aboriginal Australia. *Qual Health Res* 2016;26(5):613-25. doi: 10.1177/1049732314562891.
2.2 A purposive approach is being taken to identify 250 children from 0- 6 to take part in the study. 250 children seems a limited number to be	Thank you for your careful consideration. This has the potential to be a limitation. The aim of this study is to explore the measurement properties of this tool, rather than creating norms. Nevertheless, we

able to assess the construct validity across age. I can see this has been discussed but it is quite a big limitation of the study.	hope that we will be able to accurately describe its properties with a sample of at least 250 children. Throughout data collection, we will monitor the adequate representation of respondents to each of the categories of every item and we will continue recruitment if possible and necessary. If at the end of data collection representation won't be satisfied, we will discuss limitations of our approach as needed.
2.3 It is not clear as to whether the researchers are concerned at all about ensuring that there is a random selection of children from a representative sample of the population - rural vs urban, low SES vs high SES, children more at risk or less at risk. The validity of the tool may be less so if the selection of participants is not random. Do the authors discuss this and/or discuss the sample size which seems low for this type of study (unless conducted longitudinally).	Thank you for this thoughtful point. This study does not aim to create normative data for the ASQ-STEPS, but rather to review if the instrument items fit in the theoretical progression, and to describe the tool measurement properties when used in a large sample of Aboriginal and Torres Straits Islander children. IRT calibration and item psychometrics properties do not depend on sample characteristics. Although participant recruitment was not done systematically as it is crucial when creating norms for a measure, throughout the participant recruitment phase of this study, we ensured that children with different age ranges and families with different characteristics were enrolled.<br><br>"A volunteer sampling approach will be adopted to recruit children and their caregivers, and practitioners in the partnering sites to collect data related to objectives 1-5. Throughout the recruitment phase of this study, we will monitor enrolment to ensure that children and families with different characteristics will be enrolled."
2.4 Presently they are only collecting information on date of birth, gender, gestation, languages spoken with child, caregiver name, and caregiver relationship to the child. Will they collect information on socioeconomic status, rural vs urban, risk factors in the family, nutritional status of child, any health factors? This would be important in considering construct validity.	Thank you for your consideration. In addition to date of birth, gender, gestation, languages spoken with child and caregiver relationship to the child - which refer to the variables collected as part of standard administration of the ASQ-STEPS - we had included these additional variables in the manuscript as information we are collecting for the study: maternal level of education, number of children in the home, and socio-economic status. Maternal education is a strong predictor of nutritional status and can be used as a proxy for several variables. We will, however, be collecting information on remoteness and have now added this in the 'Analytic approach' section:

	"To test the construct validity of the ASQ-STEPS we will calculate measurement non-invariance through Differential Item Functioning. Differential Item Functioning will be assessed by child's age, sex, maternal level of education, number of children in the home, socio-economic status, ~~and~~ employment status and remoteness."
2.5 The team are conducting reliability by looking at within tool reliability. It would be helpful to understand why the team did not choose to do inter-rater or intra-rater reliability of the tool to understand how well it is functioning when used by the same or other practitioners who might be using the tool. I may have missed that the team were doing this?	Thank you for this very important point. Although inter-rater and intra-rater reliability are two important measurement properties, the implementation of this type of study requires considerable economical resources to guarantee that participants are retained in the re-testing process. We considered it important to first assess whether the adapted measure holds appropriate internal consistency and validity, including construct and content validity. Provided this study demonstrates good measurement properties and it is accepted by caregivers and practitioners, we hope to design a study to test these other important aspects of reliability.
2.7 The team might want to consider defining more carefully who the caregiver is - and will they ensure that they are consistent as to who they consider the caregiver in the study - this is likely to affect results.	Thank you for highlighting this. We have amended the section on children and caregivers to better explain the role of 'caregivers' in this context:<br>"…and have a parent, guardian or caregiver (hereafter caregiver) who consents to participate in the study. For the purpose of this study the caregiver is defined as the adult who accompanies the child and identifies as their main carer and/or has a significant connection with the child. Aboriginal and Torres Strait Islander cultural practices in family life reflect a complex and dynamic system and the responsibility of child rearing is not confined to the nuclear family [38]. Therefore, this inclusive definition acknowledges the significant role that extended family members (aunts, uncles, cousins and grandparents) play in child rearing in Aboriginal and Torres Strait Islander communities[39,40]."<br><br>Furthermore, as described in response to comment 2.8, this tool is not parent (or caregiver) completed in the way that the ASQ tools are completed, but rather is administered by interview. Therefore the child, with the support of the caregiver, completes the activity for the caregiver and practitioner to observe.

2.8 I was surprised that the tool takes 90 minutes. This seems a long time to conduct a developmental test - particularly when it is a parent reported tool. It would be good to discuss this	Thank you for identifying the need to clarify this.<br>We have made some changes to this paragraph (where the estimated time is mentioned) to highlight that, in contrast to the original ASQ tools, the ASQ-STEPS is administered by interview and is not a parent completed questionnaire. The caregiver and child are encouraged, by the practitioner, to demonstrate every item. This naturally means that the administration time is extended.<br><br>"As an interview-style assessment, caregivers are asked if they have observed their child perform a certain skill that pertains to one of the five developmental domains. The caregiver is then encouraged to support the child to demonstrate every skill, while the practitioner co-observes. On each item, the caregiver and practitioner determine if a response of "yes", "sometimes" or "not yet" is scored. Administration of the ASQ-STEPS is expected to take up to 90 minutes."<br><br>We have also added the following in the introduction, to highlight this important feature of the ASQ-STEPS:<br><br>"ASQ-STEPS measures five domains, including communication, gross motor, fine motor, personal-social and problem solving [32]. However, while the ASQ-STEPS shares the same domains, many of the same items, and the same interview style administration approach as the ASQ-TRAK…"<br><br>And we have also amended the Data Collection section on ASQ-STEPS to:<br><br>"The practitioner administers the ~~items are administered~~ ASQ-STEPS in an interview style, ~~together~~ with the caregiver, promoting a family-centred, strengths-based approach. The items ascertain a child's ability on a developmental continuum…"

	This approach, fundamental to the adaptation is also further highlighted in the Procedures section where we discuss the ASQ-STEPS training:<br><br>"The training includes content on the development of the ASQ-STEPS instrument, the family-centred, culturally responsive administration approach, scoring and providing feedback to caregivers in a strengths-based manner."
2.9 This study should be reviewed by a statistician who works in psychometrics who can review the plans for IRT analysis.	The analysis plan was designed and monitored by a team of psychometricians (Margaret Wu, Cuc Nguyen and Dan Cloney). The Analytic approach section now reads:<br><br>"Different analytical approaches will be adopted to address each of the objectives.<br>To investigate the reliability of the ASQ-STEPS we will assess ~~the internal consistency by measuring Cronbach's alpha.~~ the person separation reliability index ~~will be reported~~. This is the most important index demonstrating the extent to which the tools truly separate children by their capabilities.<br>Measurement error will be calculated to assess the uncertainty associated with measures of the sample and individual children. The internal consistency by measuring Cronbach's alpha will also be reported.<br>This study will investigate if the data collected using ASQ-STEPS fit item response models to assess whether linear measures (stages of developmental progression) can be developed to strengthen reporting. To investigate the extent to which items contribute to construct of each development domain of the ASQ-STEPS, a polytomous scored uni-dimensional item response model (IRM) [45] will be fitted to the data using the TAM package [46] of open-source statistical software program R [47]. A typical item analysis will be conducted, including assessment of classical and IRM test statistics, including Item Facility, item-rest correlation, point-biserial correlation, fit statistics (including item characteristic curves), ordering of category average abilities, item thresholds' implied order (compared to published or intended order) and dimensionality (by standardised residuals and design-implied fit) [48]. Test targeting will be reviewed by test information functions, Wright

	map, comparison between the mean of item difficulties and mean of children abilities and ~~the appropriateness of the existing basal and ceiling rules will be reviewed~~ whether there are floor or ceiling effects.<br>To test the construct validity of the ASQ-STEPS we will calculate measurement non-invariance through Differential Item Functioning. Differential Item Functioning will be assessed by child's age, sex, maternal level of education, number of children in the home, socio-economic status, employment status and remoteness.<br>Differential Item Functioning analysis will also be conducted between the Australian data and international data related to the ASQ-3: Extended to evaluate the extent to which the tools behave the same way across two populations. Latent variance-covariance matrix will also be calculated to understand the extent to which dimensions overlap."
2.10 Qualitative pieces of work - I wondered if the team were considering using any specific frameworks for understanding the themes coming from their results? Consideration of a framework for acceptability (Sekhon can be helpful) or feasibility might be helpful to provide some structure for a topic guide and possibly for some deductive thematic analysis which might also be inductive alongside. I was unclear what they then plan to do with this data in terms of the ASQ-STEPS - are they mainly looking at the way that the tool is implemented or are they looking at items within the tool?? Or other areas? E.g. feedback. Time taken, ethics etc etc….	Thank you for this useful suggestion.<br>We have elected to take an inductive approach so are not using a framework such as Sekhon. We have further clarified the approach we will take, adopting Braun and Clarke's reflexive analysis approach.<br><br>"We will adopt Braun and Clarke's reflexive thematic analysis approach [49] to analyse the qualitative data from descriptive field notes and transcripts from the practitioner and caregiver interviews. ~~Will be analysed through inductive coding of themes. Similarly, to explore face validity of the ASQ-STEPS, qualitative data from semi-structured interviews will also be analysed through inductive coding of themes.~~ The research team will transcribe field notes and audio-recordings, which will be uploaded into NVivo qualitative software [50] to facilitate coding. The IRG members and Aboriginal Research Assistants, together with the research team, will develop a coding topic guide for the initial analysis. Line by line coding of data will be conducted and initial nodes will be refined and expanded through iterative coding. Candidate ~~and~~ themes will be developed and finalised through a consensus meeting between the authors and ~~derived through an inductive process. The results will be sense checked by~~ the IRG members and Aboriginal Research Assistants."

	In table 1 we have added a comment in the 'purpose column' that indicates how the caregiver data will be utilised:<br><br>"To explore the perspectives of caregivers on the acceptability and utility of the ASQ-STEPS in more depth. This will inform the final version of the ASQ-STEPS and will also inform the approach, including training of practitioners in the implementation of the tool."

VERSION 2 - REVIEW

Reviewer 1
Name Shepherd, Carrington
Affiliation Telethon Kids Institute
Date 18-Dec-2024
COI

The authors have adequately and respectfully addressed my concerns and queries. Thank you for this. I have no further suggested modification.
